# Relationship and Sexual Quality in the Wake of COVID-19: Effects of Individual Regulatory Focus and Shared Concerns over the Pandemic

**DOI:** 10.3390/ejihpe13020035

**Published:** 2023-02-15

**Authors:** David L. Rodrigues, Rhonda N. Balzarini

**Affiliations:** 1 Department of Social and Organizational Psychology, Iscte-Instituto Universitário de Lisboa, CIS-Iscte, Av. das Forças Armadas, 1649-026 Lisboa, Portugal; 2 Department of Psychology, Texas State University, San Marcos, TX 78666, USA; 3 The Kinsey Institute, Indiana University, Bloomington, IN 47405, USA

**Keywords:** regulatory focus, relationship quality, sexual quality, sexual activity, shared concerns, COVID-19 pandemic

## Abstract

Research has shown mixed findings regarding the effect of the COVID-19 pandemic on relationship and sexual quality and activity. We argue that some of these findings might be understood considering people’s predisposition to maintain safety (i.e., prevention focus) or take risks (i.e., promotion focus), and sharing concerns with one’s partner about the pandemic. A longitudinal study (*N* = 153) tested if regulatory focus before the pandemic (November 2019) was associated with relationship quality, sexual quality, and joint sexual activity later on (June 2020) and whether these effects were moderated by shared concerns over the pandemic. Results showed that participants more focused on prevention experienced higher relationship quality later on, but also less sexual quality and less frequent joint sexual activity, when they shared fewer (vs. more) concerns with their partner. In contrast, participants more focused on promotion experienced higher relationship quality later on when they shared more (vs. less) concerns with their partner. These results indicate how individuals’ regulatory focus and shared concerns in times of crisis, such as the COVID-19 pandemic, can have downstream consequences on people’s relational and sexual dynamics. We offer insights for mental health professionals to improve psychosocial health and well-being when people are faced with critical events.

## 1. Introduction

In March 2020, the coronavirus disease (COVID-19) was declared a global pandemic. As a response, governments worldwide enforced several policies (e.g., social distancing and confinement) aimed at containing the rapid spread of new infections [1]. These policies impacted the way people felt and navigated through their daily routines and connected to others [2,3,4]. For example, schools and workplaces were shut down overnight, and leisure activities and travel were restricted for the most part. These rapid changes led to many people experiencing changes in employment (e.g., working from home), while in some cases simultaneously caring for their children and/or extended family members. Hence, people were introduced to high levels of stress and worry (e.g., about their health and the health of friends and family) and experienced both social and economic uncertainty.

These stressors had downstream consequences, with most people experiencing disruptions to their health and well-being [5], relationship dynamics [6], and sexual desire/functioning [7,8]. For example, people who experienced more pandemic-related strains (e.g., stress, loneliness) reported poorer individual outcomes, such as lower life satisfaction [9] or problems with sleep and daytime functioning [10]. Pandemic-related strains were also associated with worse relationship functioning, including less partner cohesion and relationship satisfaction [11], more conflicts [12], more avoidant conflict resolution strategies [13], and more relationship instability [14]. And yet, some of these negative effects were far from straightforward, with some people reporting no major changes since the outbreak [15,16,17], others reporting more quality in their relationship interactions [18,19], and others experiencing increases in sexual desire, sexual satisfaction, and arousal [20,21]. Indeed, when asked to think about the effects of the pandemic on their relationships, people reported more positive experiences (e.g., enjoying time together; enhanced appreciation for the partner) over negative ones (e.g., economic hardships; conflicts over the pandemic) [22,23]. We argue that some of the contradictory findings reported in the literature might be explained by differences in people’s ability to appraise the risks and benefits of a given course of action, and by having a romantic partner who shared concerns over the pandemic.

### 1.1. Perception of Risks and Benefits

There were distinct ways through which the perception of risks and the pursuit of benefits shaped personal and interpersonal functioning during the pandemic. For example, people with stronger self-control and restraint at the onset of the pandemic reported better mental health and less negative affect later on [24]. Moreover, being fearful of COVID-19 infection was associated with more health-protective behaviors [25], but also with worse diet and sleep quality [26] and negative changes in sex life [27]. Indeed, people who perceived themselves to be more susceptible to infections reported less satisfaction with their sexual activity on days they were more (but not less) worried about contracting COVID-19 [28]. In contrast, wanting to relax and connect with one’s partner during the pandemic was associated with more frequent sexual activity [29], and sexual desire was associated with more sexual exploration and sex life quality during this period [30].

One fundamental variable related to the perceptions of risks and benefits is regulatory focus. According to Regulatory Focus Theory [31,32,33], people more focused on prevention are driven by safety maintenance and seek to avoid potential losses, even at the cost of missed opportunities (e.g., they are more likely to keep a stable job instead of taking a risk with a start-up company). In contrast, people more focused on promotion are driven by pleasure and seek to obtain the most gains they can, even at the cost of losses (e.g., they are more likely to take risks in gambling when faced with the opportunity to win a jackpot). Research has already shown that regulatory focus shaped the way people felt, behaved, and related to others during the pandemic. For example, people more focused on prevention were more distressed and enacted protective behaviors more often [34], but they also reported more negative affective experiences (e.g., felt lonelier) later on [35]. Threat perceptions were also among the causes for a lowered frequency of sexual activity among people more focused on prevention [36]. Contrasting with these findings, people more focused on promotion had stronger intentions to have casual sex despite their fear of contracting COVID-19 [37] and reported less negative affective experiences and more positive affect later on [35].

### 1.2. Sharing Concerns during the Pandemic

Individual motives for security or pleasure determined how people perceived the pandemic and its potential effects on health, relationships, and sex. However, romantic partners tend to develop a common self that can act as a protective factor against adversities [38]. For example, partners who report sharing a stronger couple identity tend to use more constructive coping strategies to cope with conflicts [39]. Drawing from Interdependence [40] and Communal Coping Theories [41], sharing one’s views over potential threats (i.e., perceiving a given stressor as a common problem) helps activate adjusted coping mechanisms. Having a shared reality with the romantic partner (i.e., sharing concerns and feelings) is particularly likely to occur in times marked by negativity and uncertainty [42] and helps people perceive their partners as more responsive to their needs [43].

Research has shown that experiencing a shared reality in times of stress is important, and this is true beyond one’s relationship. For example, during the pandemic, frontline healthcare workers with a stronger shared reality felt more supported by their partner and were more satisfied in their relationship later on [44]. Romantic partners who shared concerns over the importance of joint efforts as a protection strategy during the pandemic were also likely to enact more preventive behaviors, including social distancing, wearing face masks, and washing their hands regularly [45]. Similarly, research has shown that shared reality influences relationship functioning as well. For example, the negative effect of stress on partner closeness during the pandemic was buffered when partners jointly (rather than individually) coped with stress [46]. Indeed, people who used more emotion-focused (i.e., being affectionate and intimate with the partner) and problem-focused dyadic coping (i.e., jointly discussing and finding ways to deal with an issue) reported higher relationship satisfaction and felt more secure over three weeks during the pandemic [47]. Likewise, having communal strategies to cope with the pandemic [48,49] and more supportive partner communication [50] buffered the negative association between pandemic-related stress and relationship functioning, and partners who helped each other during the pandemic experienced increases in relationship satisfaction over time [51]. In contrast, having more conflicts with the partner about the pandemic was related to less frequent sexual activity [52]. Hence, the effects of regulatory focus on relationship dynamics might have depended upon the extent to which partners shared concerns over the pandemic.

### 1.3. Current Study and Hypotheses

We launched a longitudinal study in November 2019 to examine the effects of regulatory focus in sexuality on sexual behaviors and health practices. Taking advantage of the context, we added a measurement wave in June 2020 to examine whether pre-pandemic regulatory focus determined perceptions and reactions during this global crisis. In this study, we examined if being more focused on prevention or promotion at baseline impacted relationship quality, sexual quality, and the frequency of joint sexual activity three months after COVID-19 was declared a global pandemic. We additionally examined whether some of these associations changed by having shared concerns with the partner.

People perceive and react to contextual cues differently based on their predominant prevention or promotion motives [31,32,33]. Driven by their safety maintenance motives, people more focused on prevention were more aware of health threats and enacted more protective behaviors during the pandemic, but at the same time experienced more negative affect and less sexual activity [34,35,36]. Driven by their pleasure-seeking motives, people more focused on promotion intended to take risks with casual partners during the pandemic and experienced more positive affect [35,37]. Building upon these findings, we expected people more focused on prevention before the pandemic (controlling for promotion) to experience worse relationship quality (H1a), worse sexual quality (H2a), and less frequent joint sexual activity (H3a) during its first months. In contrast, people more focused on promotion before the pandemic (controlling for prevention) should experience better relationship quality (H1b), better sexual quality (H2b), and more frequent joint sexual activity (H3b) during its first months.

As sharing concerns with the partner was beneficial for relationship functioning during the pandemic [44,51] and helped people cope with some of its negative consequences [46,47,48,49,50], we expected the effects of regulatory focus on relationship quality, sexual quality, and joint sexual activity to be moderated by shared concerns over the pandemic (H4). Specifically, we expected the negative effects of being more (vs. less) focused on prevention to be alleviated by sharing more (vs. fewer) concerns with their partner (H4a). In contrast, we expected the positive effects of being more (vs. less) focused on promotion to be enhanced by sharing more (vs. fewer) concerns (H4b).

## 2. Materials and Methods

### 2.1. Participants and Procedure

This study was part of a larger longitudinal project launched before the COVID-19 pandemic, approved by the Ethics Committee at Iscte-Instituto Universitário de Lisboa (#55/2020). Results showing the impact of regulatory focus on behavioral enactment, health, and well-being are reported elsewhere [34] and no other measures were used in previous analyses.

People over the age of 18 from the UK and the USA were invited through Clickworker to participate in a longitudinal study about their sexual attitudes and behaviors in November 2019 (baseline, T1), and for a follow-up study about sexuality in the times of COVID-19 in June 2020 (T2). Of the 384 participants who completed the survey at T1, 165 completed the survey at T2 (attrition rate: 42.97%). We excluded 12 participants who failed to complete the outcome measures. 

As shown in Table 1, participants (*N* = 153) were, on average, 34 years old, and most were from the United Kingdom (51.6%), were White (73.9%), identified as heterosexual (78.4%), identified as women (52.3%), were employed (72.5%), were living in urban areas (41.8%), were dating (64.1%), had an Associate’s or Bachelor’s degree (48.4%), and were coping on their present income (38.6%).

### 2.2. Measures

#### 2.2.1. Regulatory Focus (T1 and T2)

At T1, we used the Regulatory Focus in Sexuality scale [53] to assess prevention (three items; e.g., “Not being careful enough with my sex life has gotten me into trouble at times”) and promotion motives in sexuality (six items; e.g., “I am typically striving to fulfill my desires with my sex life”). Responses were given on 7-point scales (1 = *Not at all true of me* to 7 = *Very true of me*). Items were mean aggregated for each subscale, with higher scores indicating a greater focus on prevention (*α* = 0.81; *M* = 4.95, *SD* = 1.64) or promotion (*α* = 0.92; *M* = 4.51, *SD* = 1.58). Scores on both subscales were negatively correlated, *r*(153) = −.43, *p* < 0.001. To avoid participant fatigue, we selected the item with the highest factor loading on each subscale to be included at T2. Scores for prevention (*M* = 4.24, *SD* = 1.92) and promotion (*M* = 4.50, *SD* = 1.61) were also negatively correlated at this time point, *r*(153) = −.28, *p* < 0.001. Supporting the temporal stability of our measure, we found positive correlations across waves for prevention, *r*(153) = 0.37, *p* < 0.001, and promotion scores, *r*(153) = 0.31, *p* < 0.001 (for more details, see [34]).

#### 2.2.2. Shared Concerns over the Pandemic (T2)

We assessed the extent to which partners shared concerns over the pandemic using one item from the Love in the Time of COVID study (https://loveinthetimeofcovid.me [accessed on 23 November 2022]). Participants indicated “How much have you and your partner been on the same page (i.e., been in agreement) regarding the COVID-19 situation (e.g., view on social distancing)?” using a 4-point scale (1 = *Not at all* to 4 = *Completely*). We used raw scores (*M* = 3.01, *SD* = 0.86), with higher scores indicating higher shared concerns over the pandemic.

#### 2.2.3. Relationship Quality (T2)

We used four items from the Perceived Relationship Quality Components scale [54] to assess satisfaction, commitment, passion, and trust in the relationship during the pandemic (e.g., “How satisfied are you with your partner?”). Responses were given on 7-point scales (1 = *Not at all* to 7 = *Extremely*). Items were mean aggregated (*α* = 0.88; *M* = 5.35, *SD* = 1.28), with higher scores indicating higher relationship quality. 

#### 2.2.4. Sexual Quality (T2)

We developed two items to assess sexual quality during the pandemic. Using 5-point scales (1 = *Much less* to 5 = *Much more*), participants indicated “Compared to before the COVID-19 outbreak, my sexual desire is…” and “Compared to before the COVID-19 outbreak, my sexual satisfaction is…”. Based on their correlation, *r*(153) = 0.57, *p* < 0.001, items were mean aggregated (*M* = 3.01, *SD* = 0.96) with higher scores indicating more sexual quality.

#### 2.2.5. Joint Sexual Activity (T2)

We developed two items to assess the frequency with which participants engaged in sexual activity with their partner during the pandemic. Using 7-point scales (1 = *Not at all* to 7 = *More than once a day*), participants indicated “Since the COVID-19 outbreak, how often did you have sexual intercourse?” and “Since the COVID-19 outbreak, how often did you engage in oral sex (giving or receiving)?”. Again, items were mean aggregated (*M* = 2.86, *SD* = 1.48) based on their correlations, *r*(153) = 0.85, *p* < 0.001, with higher scores indicating more frequent joint sexual activity.

### 2.3. Analytic Plan

We tested our hypotheses by computing three linear regression models, one for each outcome variable. Specifically, we examined if pre-pandemic regulatory focus was temporally associated with relationship quality, sexual quality, and joint sexual activity seven months later (i.e., three months after COVID-19 was declared a global pandemic), and if these associations were moderated by shared concerns over the pandemic. We entered regulatory focus (at T1 and T2), shared concerns (at T2), and both interactions between regulatory focus (at T1) and shared concerns (at T2) as our predictors. All predictor variables were mean-centered before computing the interaction terms [55]. When significant interactions emerged, we computed and plotted simple slopes for people sharing fewer (−1 *SD*) or more (+1 *SD*) concerns over the pandemic [56].

## 3. Results

Results of the linear regressions are summarized in Table 2. No temporal effects emerged for relationship quality, *p* = 0.088. In contrast, we found that being more focused on prevention before the pandemic was temporally associated with poorer sexual quality, *p* = 0.044, and less frequent joint sexual activity, *p* = 0.018, during the first months of the pandemic.

**Figure 1:**
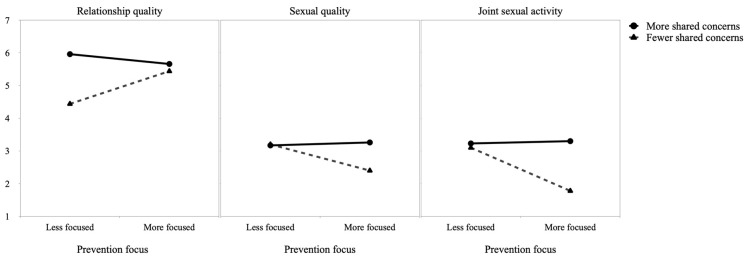
Temporal effects of prevention focus (T1) moderated by shared concerns over the pandemic (T2) on relationship quality, sexual quality, and joint sexual activity.

We also found significant interactions between prevention focus and shared concerns over the pandemic when examining relationship quality, *p* = 0.001, sexual quality, *p* = 0.009, and joint sexual activity, *p* = 0.005. As depicted in Figure 1, simple slope analyses revealed that sharing fewer concerns over the pandemic helped people more (vs. less) focused on prevention to experience better relationship quality, *p* < 0.001 (similar to people who shared more concerns, *p* = 0.396), but also to experience poorer sexual quality, *p* < 0.001 (less than people who shared more concerns, *p* < 0.001), and engage in less frequent sexual activity with their partner, *p* < 0.001 (less than people who shared more concerns, *p* < 0.001). No significant slopes emerged for people who were more (vs. less) focused on prevention and shared more concerns over the pandemic, all *p* ≥ 0.305.

**Figure 2:**
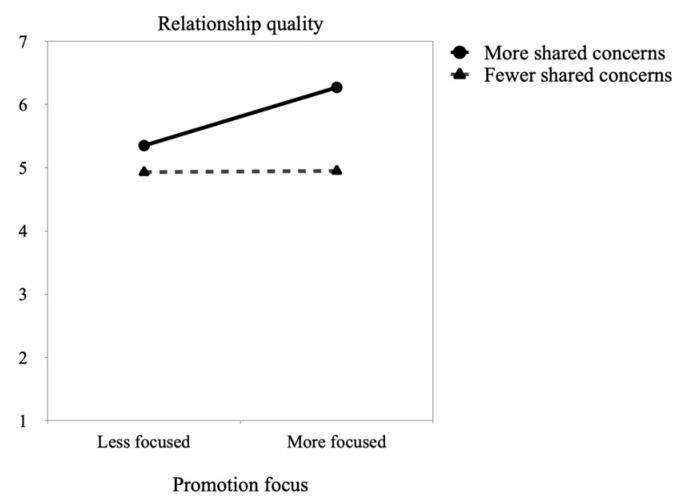
Temporal effects of promotion focus (T1) moderated by shared concerns (T2) on relationship quality.

Results also showed that being more focused on promotion before the pandemic was temporally associated with higher relationship quality during its first months, *p* = 0.021. In contrast, no temporal effects emerged for sexual quality, *p* = 0.588, or joint sexual activity, *p* = 0.122. Likewise, we only found a significant interaction between promotion focus and shared concerns over the pandemic when examining relationship quality, *p* = 0.027. No significant interactions emerged for sexual quality, *p* = 0.505, or joint sexual activity, *p* = 0.658. As depicted in Figure 2, simple slope analyses revealed that sharing more concerns over the pandemic helped people more (vs. less) focused on promotion to experience better relationship quality, *p* = 0.002 (more than people who shared fewer concerns, *p* < 0.001). The slope for people who were more (vs. less) focused on promotion and shared fewer concerns was non-significant, *p* = 0.945.

Looking at the associations between T2 variables in the linear regressions, results showed that being more focused on prevention was associated with higher relationship quality, *p* = 0.002, and sexual quality, *p* = 0.027, but not joint sexual activity, *p* = 0.450, during the first months of the pandemic. Being more focused on promotion was also associated with higher relationship quality, *p* = 0.021, sexual quality, *p* = 0.004, and joint sexual activity, *p* < 0.001. Exploratory analyses showed that the interactions between regulatory focus and shared concerns (both at T2) were not significantly associated with relationship quality, both *p* ≥ 0.412, sexual quality, both *p* ≥ 0.739, and joint sexual activity, both *p* ≥ 0.605, when entered as additional predictor variables in the linear regression models.

## 4. Discussion

Using longitudinal data, we examined the implications of pre-pandemic predominant regulatory focus on relationship and sexual quality and activity in the wake of the COVID-19 global pandemic, and whether these effects were moderated by sharing concerns with the partner about its foreseeable consequences. Partially supporting our hypotheses, results of the direct temporal associations showed that people more focused on prevention reported a worsening of their sexual quality (H2a) and decreases in joint sexual activity (H3a), whereas people more focused on promotion only reported an improvement in their relationship quality (H1b). Also partially supporting our hypotheses, sharing concerns with the partner over the pandemic resulted in different outcomes. However, it did not alleviate the expected negative effects of being more focused on prevention (H4a). Instead, sharing fewer (but not more) concerns was beneficial for the experience of relationship quality among people more focused on prevention. On the other hand, sharing more (but not fewer) concerns with the partner over the pandemic resulted in an enhancement of relationship quality for people more focused on promotion, as we expected (H4b).

Our findings are generally aligned with research conducted during the pandemic, linking not only prevention focus with negative affective and sexual experiences [35,36], but also promotion focus with positive affective experiences [35]. More importantly, our findings also highlight the nuanced effects of regulatory focus when examining how people behaved in their relationships at the onset of the pandemic. On the one hand, the findings that sexual quality and activity (but not relationship quality) decreased because of prevention focus suggest that these people prioritized their physical health. Indeed, research [34] has shown that being more focused on prevention led people to become more worried about contracting COVID-19 and enact preventive behaviors more often. Despite their efforts to protect themselves and others, however, being more focused on prevention also increased perceptions of risk and, in turn, worse physical health. Considering our current findings, we believe that people more focused on prevention, who struggled to cope with high levels of induced stress, might have sought their partner’s support to be understood and reduce their negative affect levels. Having a partner who did not necessarily share similar concerns over the pandemic (e.g., having different perceptions about its severity) might have helped to decrease their negative experiences (e.g., rethink the risk levels of the pandemic) and contribute to increasing relational well-being. However, this benefit was not reflected in their sex lives. In fact, sharing fewer concerns worsened their sexual quality and contributed to a decreased frequency of sexual activity. Considering that sexual responses are disrupted by stressful experiences [57,58] and by disease avoidance motives [59], people more focused on prevention might have been more prone to disruptions in their sex life particularly when they felt their partner was not worried about the severity of the pandemic.

On the other hand, the finding that relationship quality (but not sexual quality or activity) improved because of promotion focus suggests that these people prioritized their relational well-being. Indeed, research has shown that people more focused on promotion believe that relationship growth is particularly important [60], use constructive conflict resolution styles [61], and tend to activate relationship protection strategies [62]. This is particularly evident when people are more committed to their relationship, evidencing an overlap between individual and relational growth motives in this situation [40,63]. Arguably, these people had lesser concerns about the potential risks of the pandemic, and having a partner with similar views might have helped them feel understood and supported, which enhanced their relationship quality. However, this was not reflected in their sex lives. Even though this finding was particularly puzzling given the motivational priorities of having a promotion focus (i.e., pleasure over safety), we cannot rule out the possibility that some people more focused on promotion experienced improvements, whereas others experienced disruptions, in their sex lives.

### Limitations and Future Research

We must acknowledge some limitations to our study. We focused our analysis on a broad assessment of shared concerns over the potential implications of the pandemic and failed to assess intrapersonal factors (e.g., personal history with COVID-19 infection) or other interpersonal and contextual dynamics (e.g., family conditions; need to expose oneself to risks based on work demands) that could have helped explain some of the non-significant results. Likewise, our study focused on the participants’ perspective of their relationship, and without their partner’s responses, we cannot determine the actual perspective of the partner over the pandemic. Researchers who collected dyadic data during the pandemic and used similar constructs might consider testing our hypotheses, and having additional variables will help explain our pattern of results to a greater extent. Including both partners would additionally provide insights into whether having a match or mismatch between partners’ regulatory focus (or security vs. pleasure motives) is determinant for relationship and sexual quality and activity. It would be interesting to understand if individual motives interacted with different types of concerns (e.g., health, family, or work concerns) and other factors and dynamics to change people’s experiences and reactions to the pandemic. For example, research has shown that people more focused on promotion experience more sexual pleasure and enact riskier sexual practices, but they also get tested for sexually transmitted infections more often [64,65]. Hence, getting both partners tested for COVID-19 more often might have been one of the conditions under which people more focused on promotion experienced improvements in their sex lives. We also focused our analysis on the first months of the pandemic. We found evidence of temporal stability in regulatory focus. However, the correlations between time points were moderate in magnitude and indicate that people may have adjusted their perceptions and behaviors in response to the pandemic context. Indeed, we are unsure of whether or how the impact of regulatory focus and shared concerns unfolded over a longer period. Arguably, motivations to avoid or take risks (i.e., regulatory focus) changed as a function of social policies in effect or based on contextual changes. For instance, people more focused on prevention might have been less anxious or threatened as the number of infections started to decrease, as social confinement policies were revised or became less strict, or as vaccines were made available. It would be interesting to understand if individual motives interacted with these broader contextual factors to change not only how people recalled their experiences, but also how they perceive the current situation and the severity of risks, and how they anticipate their reactions if faced with a new pandemic situation.

## 5. Conclusions

Our longitudinal study offered relevant insights into the COVID-19 pandemic and how the pandemic and people’s motivational tendencies have influenced their relationships. More specifically, this research shows for the first time the effects of regulatory focus and shared concerns in determining how people perceive and behave in their romantic relationships when faced with a global pandemic. On a broader level, our results can be used to inform the development of awareness campaigns to educate people about times of crisis and the consequences for their individual and relational health and well-being. On a more specific level, our results can also help professionals (e.g., therapists) communicate with their patients and understand their reactions, to help them deal with current problems (e.g., relational or sexual consequences of the COVID-19 pandemic) or develop relationship strategies for future anxiety-provoking events (e.g., sharing specific concerns that may be triggering negative experiences).

## Figures and Tables

**Table 1:** Sociodemographic characteristics.

	*M* (*SD*)	*n* (%)
*Age* (range: 18–50)	34.16 (7.55)	
*Country*		
United Kingdom		79 (51.6)
United States of America		74 (48.4)
*Race/Ethnicity*		
White		113 (73.9)
Black		15 (9.8)
Asian		8 (5.2)
Mixed-race		8 (5.2)
Latinx		3 (2.0)
Arab		1 (0.7)
Indian		1 (0.7)
Prefer not to answer		4 (2.6)
*Sexual orientation*		
Heterosexual		120 (78.4)
Bisexual		20 (13.1)
Lesbian/Gay		6 (3.9)
Other (e.g., pansexual, queer)		4 (2.6)
Prefer not to answer		3 (2.0)
*Gender*		
Female		80 (52.3)
Male		72 (47.1)
Prefer not to answer		1 (0.7)
*Occupation*		
Employed		111 (72.5)
Unemployed		22 (14.4)
Student and working		11 (7.2)
Student		5 (3.3)
Stay-at-home parent		3 (2.0)
Retired		1 (0.7)
*Residence*		
Urban areas		64 (41.8)
Suburban areas		63 (41.2)
Rural areas		26 (17.0)
*Relationship status*		
Dating		98 (64.1)
Married/Civil union		55 (35.9)
*Education*		
High school		47 (30.7)
Associate’s/Bachelor’s degree		74 (48.4)
Master’s degree		30 (19.6)
Doctorate’s degree		2 (1.3)
*Perceived socioeconomic status*		
Very difficult/difficult on present income		55 (35.9)
Coping on present income		59 (38.6)
Comfortable/Very comfortable on present income		39 (25.5)

**Table 2:** Interaction between regulatory focus (T1) and shared concerns over the pandemic (T2) on relationship quality, sexual quality, and joint sexual activity.

	Relationship Quality (*R*^2^ = 0.33)	Sexual Quality (*R*^2^ = 0.12)	Joint Sexual Activity (*R*^2^ = 0.20)
	*b* (*SE*)	*b* (*SE*)	*b* (*SE*)
Prevention focus (T1)	0.11 (0.06)	−0.11 * (0.05)	−0.19 * (0.08)
Promotion focus (T1)	0.15 * (0.06)	−0.03 (0.05)	−0.12 (0.08)
Shared concerns over the pandemic (T2)	0.51 *** (0.10)	0.24 ** (0.09)	0.48 *** (0.13)
Prevention (T1) x Shared concerns (T2)	−0.23 *** (0.07)	0.16 ** (0.06)	0.25 ** (0.09)
Fewer shared concerns	0.30 *** (0.08)	−0.24 *** (0.07)	−0.40 *** (0.10)
More shared concerns	−0.09 (0.09)	0.03 (0.08)	0.02 (0.11)
Promotion (T1) x Shared concerns (T2)	0.17 * (0.07)	0.04 (0.06)	0.04 (0.09)
Fewer shared concerns	0.01 (0.09)	-	-
More shared concerns	0.29 ** (0.09)	-	-
Prevention focus (T2)	0.15 ** (0.05)	0.10 * (0.04)	0.05 (0.06)
Promotion focus (T2)	0.14 * (0.06)	0.15 ** (0.05)	0.32 *** (0.07)

Note. *b* = unstandardized coefficients, *SE* = standard error. Significant results are in boldface. Collinearity statistics, as represented by the Variance Inflation Factor (VIF), revealed the absence of multicollinearity. VIFs ranged from 1.01 to 1.44 in all regressions. * *p* ≤ 0.050. ** *p* ≤ 0.010. *** *p* ≤ 0.001.

## Data Availability

The data presented in this study are openly available on the Open Science Framework at [https://doi.org/10.17605/osf.io/h35mf].

## References

[1] Abouk R., Heydari B. (2021). The Immediate Effect of COVID-19 Policies on Social-Distancing Behavior in the United States. Public Health Rep..

[2] Maison D., Jaworska D., Adamczyk D., Affeltowicz D. (2021). The Challenges Arising from the COVID-19 Pandemic and the Way People Deal with Them. A Qualitative Longitudinal Study. PLoS ONE.

[3] Möhring K., Naumann E., Reifenscheid M., Wenz A., Rettig T., Krieger U., Friedel S., Finkel M., Cornesse C., Blom A.G. (2021). The COVID-19 Pandemic and Subjective Well-Being: Longitudinal Evidence on Satisfaction with Work and Family. Eur. Soc..

[4] Zoppolat G., Righetti F., Balzarini R.N., Alonso-Ferres M., Urganci B., Rodrigues D.L., Debrot A., Wiwattanapantuwong J., Dharma C., Chi P. (2022). Relationship Difficulties and “Technoference” during the COVID-19 Pandemic. J. Soc. Pers. Relatsh..

[5] Ettman C.K., Cohen G.H., Abdalla S.M., Sampson L., Trinquart L., Castrucci B.C., Bork R.H., Clark M.A., Wilson I., Vivier P.M. (2022). Persistent Depressive Symptoms during COVID-19: A National, Population-Representative, Longitudinal Study of U.S. Adults. Lancet Reg. Health Am..

[6] Pietromonaco P.R., Overall N.C. (2022). Implications of Social Isolation, Separation, and Loss during the COVID-19 Pandemic for Couples’ Relationships. Curr. Opin. Psychol..

[7] Delcea C., Chirilă V.-I., Săuchea A.-M. (2021). Effects of COVID-19 on Sexual Life—A Meta-Analysis. Sexologies.

[8] Balzarini R.N., Muise A., Zoppolat G., Gesselman A.N., Lehmiller J.J., Garcia J.R., Slatcher R.B., Mark K.P. (2022). Sexual Desire in the Time of COVID-19: How COVID-Related Stressors Are Associated with Sexual Desire in Romantic Relationships. Arch. Sex Behav..

[9] Temiz Z.T., Elsharnouby E. (2022). Relationship Satisfaction and Well-Being during the COVID-19 Pandemic: Examining the Associations with Interpersonal Emotion Regulation Strategies. Cogn. Ther. Res..

[10] Zerbini G., Taflinger S., Reicherts P., Kunz M., Sattler S. (2022). Perceived Risk of COVID-19 Exposure and Poor COVID-19 Prognosis Impair Sleep: The Mediating and Moderating Roles of COVID-19-Related Anxiety and Knowledge. J. Sleep Res..

[11] Overall N.C., Chang V.T., Pietromonaco P.R., Low R.S.T., Henderson A.M.E. (2021). Partners’ Attachment Insecurity and Stress Predict Poorer Relationship Functioning during COVID-19 Quarantines. Soc. Psychol. Personal. Sci..

[12] Balzarini R.N., Muise A., Zoppolat G., Di Bartolomeo A., Rodrigues D.L., Alonso-Ferres M., Urganci B., Debrot A., Pichayayothin N.B., Dharma C. (2022). Love in the Time of COVID: Perceived Partner Responsiveness Buffers People from Lower Relationship Quality Associated with COVID-Related Stressors. Soc. Psychol. Personal. Sci..

[13] Li Y., Samp J.A. (2021). The Impact of the COVID-19 Pandemic on Same-Sex Couples’ Conflict Avoidance, Relational Quality, and Mental Health. J. Soc. Pers. Relatsh..

[14] Ogan M.A., Monk J.K., Kanter J.B., Proulx C.M. (2021). Stress, Dyadic Coping, and Relationship Instability during the COVID-19 Pandemic. J. Soc. Pers. Relatsh..

[15] Cénat J.M., Farahi S.M.M.M., Dalexis R.D., Darius W.P., Bekarkhanechi F.M., Poisson H., Broussard C., Ukwu G., Auguste E., Nguyen D.D. (2022). The Global Evolution of Mental Health Problems during the COVID-19 Pandemic: A Systematic Review and Meta-Analysis of Longitudinal Studies. J. Affect. Disord..

[16] Mitchell K.R., Shimonovich M., Bosó Pérez R., Dema E., Clifton S., Riddell J., Copas A.J., Tanton C., Macdowall W., Bonell C. (2023). Initial Impacts of COVID-19 on Sex Life and Relationship Quality in Steady Relationships in Britain: Findings from a Large, Quasi-Representative Survey (Natsal-COVID). J. Sex Res..

[17] Robinson E., Sutin A.R., Daly M., Jones A. (2022). A Systematic Review and Meta-Analysis of Longitudinal Cohort Studies Comparing Mental Health before versus during the COVID-19 Pandemic in 2020. J. Affect. Disord..

[18] Bevan J.L., Murphy M.K., Lannutti P.J., Slatcher R.B., Balzarini R.N. (2023). A Descriptive Literature Review of Early Research on COVID-19 and Close Relationships. J. Soc. Pers. Relatsh..

[19] Toldam N.E., Graugaard C., Meyer R., Thomsen L., Dreier S., Jannini E.A., Giraldi A. (2022). Sexual Health during COVID-19: A Scoping Review. Sex. Med. Rev..

[20] Panzeri M., Ferrucci R., Cozza A., Fontanesi L. (2020). Changes in Sexuality and Quality of Couple Relationship during the COVID-19 Lockdown. Front. Psychol..

[21] Wignall L., Portch E., McCormack M., Owens R., Cascalheira C.J., Attard-Johnson J., Cole T. (2021). Changes in Sexual Desire and Behaviors among UK Young Adults during Social Lockdown Due to COVID-19. J. Sex Res..

[22] Günther-Bel C., Vilaregut A., Carratala E., Torras-Garat S., Pérez-Testor C. (2020). A Mixed-Method Study of Individual, Couple, and Parental Functioning during the State-Regulated COVID-19 Lockdown in Spain. Fam. Process.

[23] Holmberg D., Bell K.M., Cadman K. (2022). Now for the Good News: Self-Perceived Positive Effects of the First Pandemic Wave on Romantic Relationships Outweigh the Negative. J. Soc. Pers. Relatsh..

[24] Martínez-Martí M.L., Theirs C.I., Pascual D., Corradi G. (2020). Character Strengths Predict an Increase in Mental Health and Subjective Well-Being over a One-Month Period during the COVID-19 Pandemic Lockdown. Front. Psychol..

[25] Šuriņa S., Martinsone K., Perepjolkina V., Kolesnikova J., Vainik U., Ruža A., Vrublevska J., Smirnova D., Fountoulakis K.N., Rancans E. (2021). Factors Related to COVID-19 Preventive Behaviors: A Structural Equation Model. Front. Psychol..

[26] Keng S.-L., Stanton M.V., Haskins L.B., Almenara C.A., Ickovics J., Jones A., Grigsby-Toussaint D., Agostini M., Bélanger J.J., Gützkow B. (2022). COVID-19 Stressors and Health Behaviors: A Multilevel Longitudinal Study across 86 Countries. Prev. Med. Rep..

[27] Rodrigues D.L., Lehmiller J.J. (2022). COVID-19 and Sexual Desire: Perceived Fear Is Associated with Enhanced Relationship Functioning. J. Sex Res..

[28] Hicks L.L., Meltzer A.L., French J.E., Altgelt E.E., Turner J.A., McNulty J.K. (2022). Perceptions of Infectability to Disease Moderate the Association between Daily Concerns about Contracting COVID-19 and Satisfaction with Sex. Arch. Sex. Behav..

[29] Ballester-Arnal R., Nebot-Garcia J.E., Ruiz-Palomino E., Giménez-García C., Gil-Llario M.D. (2021). “INSIDE” Project on Sexual Health in Spain: Sexual Life during the Lockdown Caused by COVID-19. Sex Res Soc Policy.

[30] Lehmiller J.J., Garcia J.R., Gesselman A.N., Mark K.P. (2021). Less Sex, but More Sexual Diversity: Changes in Sexual Behavior during the COVID-19 Coronavirus Pandemic. Leis. Sci..

[31] Scholer A.A., Higgins E.T. (2012). Too Much of a Good Thing? Trade-Offs in Promotion and Prevention Focus. The Oxford Handbook of Human Motivation.

[32] Higgins E.T., Scott R.A., Buchmann M.C., Kosslyn S.M. (2015). Regulatory Focus Theory. Emerging Trends in the Social and Behavioral Sciences: An Interdisciplinary, Searchable, and Linkable Resource.

[33] Zou X., Scholer A.A. (2016). Motivational Affordance and Risk-Taking across Decision Domains. Pers. Soc. Psychol. Bull.

[34] Rodrigues D.L., Lopes D., Balzarini R.N. (2022). Having a Prevention Regulatory Focus Longitudinally Predicted Distress and Health-Protective Behaviours during the COVID-19 Pandemic. Stress Health.

[35] Rodrigues D.L., Zoppolat G., Balzarini R.N., Slatcher R.B. (2022). Security Motives and Negative Affective Experiences during the Early Months of the COVID-19 Pandemic. Psychol. Health.

[36] Rodrigues D.L., Balzarini R.N., Zoppolat G., Slatcher R.B. (2022). Motives for Security and Sexual Activity among Single Individuals at the Onset of the COVID-19 Pandemic. Psychol. Sex..

[37] Rodrigues D.L. (2022). Regulatory Focus and Perceived Safety with Casual Partners: Implications for Perceived Risk and Casual Sex Intentions during the COVID-19 Pandemic. Psychol. Sex..

[38] Aron A., Lewandowski G., Branand B., Mashek D., Aron E. (2022). Self-Expansion Motivation and Inclusion of Others in Self: An Updated Review. J. Soc. Pers. Relatsh..

[39] Walsh C.M., Neff L.A. (2018). We’re Better When We Blend: The Benefits of Couple Identity Fusion. Self Identity.

[40] Rusbult C., Arriaga X., Agnew C., Fletcher G., Clark M. (2001). Interdependence in Close Relationships. Blackwell Handbook of Social Psychology: Interpersonal Processes.

[41] Afifi T.D., Hutchinson S., Krouse S. (2006). Toward a Theoretical Model of Communal Coping in Postdivorce Families and Other Naturally Occurring Groups. Commun. Theory.

[42] Bar-Shachar Y., Bar-Kalifa E. (2021). Responsiveness Processes and Daily Experiences of Shared Reality among Romantic Couples. J. Soc. Pers. Relatsh..

[43] Rossignac-Milon M., Bolger N., Zee K.S., Boothby E.J., Higgins E.T. (2021). Merged Minds: Generalized Shared Reality in Dyadic Relationships. J. Personal. Soc. Psychol..

[44] Enestrom M.C., Lydon J.E. (2021). Relationship Satisfaction in the Time of COVID-19: The Role of Shared Reality in Perceiving Partner Support for Frontline Health-Care Workers. J. Soc. Pers. Relatsh..

[45] Starks T.J., Bosco S.C., Doyle K.M., Revenson T.A. (2022). Partners’ Consensus about Joint Effort and COVID-19 Prevention among Sexual Minority Men. Arch. Sex. Behav..

[46] Salo K.I., Pauw L.S., Echterhoff G., Milek A. (2022). Daily Stress, Closeness, and Coping in Romantic Relationships during COVID-19-Related Lockdown: An Experience-Sampling Study. Couple Fam. Psychol. Res. Pract..

[47] Vedelago L., Balzarini R.N., Fitzpatrick S., Muise A. (2022). Tailoring Dyadic Coping Strategies to Attachment Style: Emotion-Focused and Problem-Focused Dyadic Coping Differentially Buffer Anxiously and Avoidantly Attached Partners. J. Soc. Pers. Relatsh..

[48] Jones H.E., Yoon D.B., Theiss J.A., Austin J.T., Lee L.E. (2021). Assessing the Effects of Covid-19 on Romantic Relationships and the Coping Strategies Partners Use to Manage the Stress of a Pandemic. J. Fam. Commun..

[49] Randall A.K., Leon G., Basili E., Martos T., Boiger M., Baldi M., Hocker L., Kline K., Masturzi A., Aryeetey R. (2022). Coping with Global Uncertainty: Perceptions of COVID-19 Psychological Distress, Relationship Quality, and Dyadic Coping for Romantic Partners across 27 Countries. J. Soc. Pers. Relatsh..

[50] Martin L.N., Giff S.T., Ribeiro S., Fyffe S., Renshaw K.D. (2022). Changes in Relationship Quality in the COVID-19 Pandemic: Associations with Pandemic Stressors and Couple Communication. Am. J. Fam. Ther..

[51] Williamson H.C. (2020). Early Effects of the COVID-19 Pandemic on Relationship Satisfaction and Attributions. Psychol. Sci..

[52] Luetke M., Hensel D., Herbenick D., Rosenberg M. (2020). Romantic Relationship Conflict Due to the COVID-19 Pandemic and Changes in Intimate and Sexual Behaviors in a Nationally Representative Sample of American Adults. J. Sex Marital. Ther..

[53] Rodrigues D.L., Lopes D., Pereira M., Prada M., Garrido M.V. (2019). Motivations for Sexual Behavior and Intentions to Use Condoms: Development of the Regulatory Focus in Sexuality Scale. Arch. Sex. Behav..

[54] Fletcher G.J.O., Simpson J.A., Thomas G. (2000). The Measurement of Perceived Relationship Quality Components: A Confirmatory Factor Analytic Approach. Pers. Soc. Psychol. Bull..

[55] Aiken L.S., West S.G. (1991). Multiple Regression: Testing and Interpreting Interactions.

[56] Preacher K.J., Curran P.J., Bauer D.J. (2006). Computational Tools for Probing Interactions in Multiple Linear Regression, Multilevel Modeling, and Latent Curve Analysis. J. Educ. Behav. Stat..

[57] Bodenmann G., Atkins D.C., Schär M., Poffet V. (2010). The Association between Daily Stress and Sexual Activity. J. Fam. Psychol..

[58] Hamilton L.D., Julian A.M. (2014). The Relationship between Daily Hassles and Sexual Function in Men and Women. J. Sex Marital. Ther..

[59] Gruijters S.L.K., Tybur J.M., Ruiter R.A.C., Massar K. (2016). Sex, Germs, and Health: Pathogen-Avoidance Motives and Health-Protective Behaviour. Psychol. Health.

[60] Cortes K., Scholer A.A., Kohler A., Cavallo J.V. (2018). Perceiving Relationship Success through a Motivational Lens: A Regulatory Focus Perspective. Pers. Soc. Psychol. Bull..

[61] Rodrigues D.L., Huic A., Lopes D., Kumashiro M. (2019). Regulatory Focus in Relationships and Conflict Resolution Strategies. Personal. Individ. Differ..

[62] Rodrigues D.L., Lopes D., Kumashiro M. (2017). The “I” in Us, or the Eye on Us? Regulatory Focus, Commitment and Derogation of an Attractive Alternative Person. PLoS ONE.

[63] Aron A., Mashek D., Aron E., Mashek D., Aron A. (2004). Closeness as Including Other in the Self. Handbook of Closeness and Intimacy.

[64] Evans-Paulson R., Widman L., Javidi H., Lipsey N. (2022). Is Regulatory Focus Related to Condom Use, STI/HIV Testing, and Sexual Satisfaction?. J. Sex Res..

[65] Rodrigues D.L., Lopes D., Carvalho A.C. (2022). Regulatory Focus and Sexual Health: Motives for Security and Pleasure in Sexuality Are Associated with Distinct Protective Behaviors. J. Sex Res..

